# Is Wildlife Fertility Control Always Humane?

**DOI:** 10.3390/ani5040398

**Published:** 2015-10-21

**Authors:** Jordan O. Hampton, Timothy H. Hyndman, Anne Barnes, Teresa Collins

**Affiliations:** College of Veterinary Medicine, Murdoch University, 90 South Street, Murdoch 6150, Australia; E-Mails: t.hyndman@murdoch.edu.au (T.H.H.); a.barnes@murdoch.edu.au (A.B.); t.collins@murdoch.edu.au (T.C.)

**Keywords:** behaviour, capture, endocrinology, fertility, immunocontraception, physiology, reproduction, sterilization, welfare, wildlife

## Abstract

**Simple Summary:**

There are various fertility control methods (modalities) currently available that aim to reduce the abundance of problematic free-ranging mammalian wildlife. Here, we propose that dissimilarities in the mechanism of action indicate these methods produce great variation in animal welfare outcomes. We present a framework to assist managers in minimising animal welfare risks.

**Abstract:**

Investigation of fertility control techniques to reduce reproductive rates in wildlife populations has been the source of much research. Techniques targeting wildlife fertility have been diverse. Most research into fertility control methods has focused upon efficacy, with few studies rigorously assessing animal welfare beyond opportunistic anecdote. However, fertility control techniques represent several very different mechanisms of action (modalities), each with their own different animal welfare risks. We provide a review of the mechanisms of action for fertility control methods, and consider the role of manipulation of reproductive hormones (“endocrine suppression”) for the long-term ability of animals to behave normally. We consider the potential welfare costs of animal manipulation techniques that are required to administer fertility treatments, including capture, restraint, surgery and drug delivery, and the requirement for repeated administration within the lifetime of an animal. We challenge the assumption that fertility control modalities generate similar and desirable animal welfare outcomes, and we argue that knowledge of reproductive physiology and behaviour should be more adeptly applied to wild animal management decisions. We encourage wildlife managers to carefully assess long-term behavioural risks, associated animal handling techniques, and the importance of positive welfare states when selecting fertility control methods as a means of population control.

## 1. Introduction

Animal welfare has been steadily gaining importance and society now recognises it has an obligation to consider the welfare of all animals, including free-ranging species that are perceived to be overabundant or pests. As community and ethical opposition to the killing of animals has risen, especially for charismatic species (e.g., feral horses (*Equus caballus*); [1]), more focus has been devoted to non-lethal methods, particularly fertility control [2]. Fertility control methods have often been grouped together and described using terms such as “humane” [3,4,5], or “benign” [6,7]. These are not universal opinions though, as some authors have urged consideration of potential animal welfare impacts [8,9], including recognition that the welfare consequences of different methods may vary considerably [10].

Despite the importance of animal welfare, there are other aspects of fertility control methods that cannot be ignored. Efficacy will always need to be considered because if a method is chosen that has a reduced welfare impact on the individual, but does not achieve its animal management goals, then arguably doing nothing at all would have resulted in better animal welfare outcomes [11]. The overall welfare impacts of fertility control techniques should also be considered alongside the impacts of other control tools that may be considered as an alternative [12]. In any context, once an “optimal” decision has been made, it should not be blindly extrapolated to all species for all circumstances. With this in mind, there are large gaps in the published literature as most research has focused on the outcomes of fertility control programs in terms of reproductive output (its efficacy). Much less research has examined how each method induces decreased reproductive output, and at what cost to the animal. This evolution of research into wildlife fertility control methods appears to be tracking the same course that investigations into the use of lethal toxins for wildlife control followed, where efficacy studies preceded assessments of welfare impacts [13].

### Aims of This Review

Once it has been decided that a fertility control program will be employed, there is considerable uncertainty when choosing a specific fertility control product or technique. Several authors have attempted to address the broad side-effects associated with fertility control of wildlife, including animal welfare impacts [9,14,15], but the relative risks of each fertility control method has so far escaped critical review. However, the American Zoo and Aquarium (AZA) Contraception Advisory Group has provided several articles examining the relative risks of fertility control modalities relevant to collection animals (e.g., [16]). Equivalent critical reviews have been performed for the manipulation of reproductive hormones in human medicine [17], highlighting that divergent properties of fertility control methods are related to dissimilarities in mechanisms of action, and ultimately produce variable clinical consequences.

This review attempts to demonstrate the differences between broad categories of fertility control techniques, with a view to facilitating future selection of practices. Fertility control has been applied to non-mammalian wildlife (such as birds; [18]) but this review will restrict its examination to mammalian wildlife. The range of fertility control approaches used to manage mammalian wildlife is discussed, with a focus on mechanisms of action, the importance of maintaining natural behaviour and the need to assess all stages of animal manipulation involved in administering required treatments. We suggest principles to advance the scientific rigour behind the selection of fertility control techniques toward a “best practice” model to maximise animal welfare outcomes.

The central aim of this study is to specifically provide a framework for assessing animal welfare risks for various fertility control methods for mammalian wildlife. It cannot be disputed that efficacy and financial costs are important considerations when management decisions are being made, however, these issues have been thoroughly addressed by other reviews [2] and so will not be discussed in detail in this study. The aims of this study are not to compare fertility control with lethal methods, and not to assess side-effects of fertility control methods as a whole. This review explicitly examines animal welfare risks specific to each class of fertility control method and presents a framework for selecting fertility control methods that minimise animal welfare risks.

## 2. Fertility Control Methods Currently Used

A wide diversity of methods has been applied to wildlife fertility control over several decades. Table 1 lists the most commonly applied fertility control approaches in free-ranging mammals. Fertility control methods vary greatly in the mechanism of action of the treatment applied, as well as the administration method used to deliver them to animals (e.g., surgery *versus* remote injection or “darting”). Table 1 also illustrates that within individual species (e.g., white-tailed deer (*Odocoileus virginianus*)), multiple fertility control methods, representing several mechanisms of action, have been employed. These methods, and many more, have previously been reviewed in detail [2,15]. Several approaches to classifying the various fertility control methods have been suggested (e.g., physical *versus* chemical), but given the inextricable association between any method’s mechanism of action and its effect, we argue that classification according to mechanism of action is the most logical approach.

### 2.1. Mechanisms of Action

Owing to the diversity of fertility control techniques currently available (Table 1; [2]), an appreciation of welfare impacts is not possible unless focus is first placed upon mechanisms of action. “Mechanistic” approaches have been central to rationalising and standardising the use of other wildlife management techniques. For example, rationalisation of a vast array of trade and colloquial names has been possible for toxins [13] and kill-traps [19] that are subjected to welfare assessment. All effects are produced by a mechanism of action, and dissimilarities in these processes in fertility control need to be understood to avoid misunderstanding [20]. For this reason we recommend the use of the term fertility control modalities to highlight the importance of considering how a method works and the long term welfare outcome at the individual animal level, rather than simply considering efficacy at a population level.

**Table 1:** Wildlife fertility control modalities that have been trialled, categorised according to their mechanism of action.

Class	Technique	Route	Description	Duration of Effect	Examples of Products	Sex Sterilized	Example Application	
							Species	Reference
Endocrine suppression	Castration (gonadectomy)	Surgical	Removal of testicles	Permanent	N/A	Male	Eastern grey kangaroos (*Macopus giganteus*)	[21]
	Ovariectomy/ovariohysterectomy (gonadectomy)	Surgical	Removal of ovaries +/− uterus	Permanent	N/A	Female	Brushtail possums (*Trichosurus vulpecula*)	[22]
	GnRH agonism	Implant	Binds to GnRH receptors	1–2 years	Deslorelin^®^	Female	Tammar wallabies (*Macropud eugenii*)	[23,24]
					Suplorelin^®^	Male		
	GnRH immunocontraception	Injection	Induced immunity to GnRH	>3 years	GonaCon^®^	Female	Wild boar (*Sus scrofa*)	[25]
						Male		
	Synthetic testosterone	Injection	Suppressed spermatogenesis	Temporary	N/A	Male	Wild horses (*Equus caballus*)	[26]
	Synthetic progestins	Implant	Prevention of ovulation	3–5 years	Norplant^®^	Female	Koalas (*Phascolarctos cinereus*)	[27]
	Synthetic prostaglandin	Implant/biobullet	Pregnancy termination	Temporary	N/A	Female	White-tailed deer (*Odocoileus virginianus*)	[28]
	Synthetic oestrogen	Oral bait	Prevention of implantation	Temporary	N/A	Female	White-tailed deer (*O. virginianus*)	[29]
	Synthetic oestrogen	Implant	Prevention of implantation	Temporary	N/A	Female	White-tailed deer (*O. virginianus*)	[30]
Physical non-endocrine	Vasectomy	Surgical	Ligation of vas deferens	Permanent	N/A	Male	Eastern grey kangaroos (*M. giganteus*)	[21]
	Epididymectomy	Surgical	Removal of epididymis	Permanent	N/A	Male	Feral goats (*Capra hircus*)	[31]
	Tubal ligation	Surgical	Ligation of oviducts	Permanent	N/A	Female	White-tailed deer (*O. virginianus*)	[32]
	Oviduct “webbing”	Surgical	Removal of oviducts	Permanent	N/A	Female	Feral cattle (*Bos taurus*)	[33]
	Intra-testicular injection	Injection	Sclerosis of testicles	Permanent	Neutersol^®^	Male	American black bears (*Ursus americanus*)	[34]
	Intra-uterine devices	Implant	Endometrium irritation	Temporary	N/A	Female	Wild horses (*E. caballus*)	[35]
Chemical non-endocrine	Zona pellucida immunocontraception	Injection	Induced immunity to ovum proteins	>3 years	ZonaStat-H^®^	Female	Tule elk (*Cervus elaphus nannodes*)	[36]
					SpayVac^®^			

### 2.2. Broad Classes of Fertility Control Modalities

In order to clarify the modalities for each fertility control agent, the following broad classes should be adopted: endocrine suppression methods, physical non-endocrine methods, and chemical non-endocrine methods (Table 1).

“Endocrine suppression” [37,38] (or endocrine disruption) methods alter the actions of naturally occurring reproductive hormones. Endocrine processes may be targeted centrally, depressing all reproductive hormonal activity (e.g., gonadotrophin-releasing-hormone (GnRH) agonists) or peripherally (e.g., progestin antagonists). Such is the complex nature of reproductive endocrine feedback processes that all supplementation, agonism, antagonism, or removal of endocrine hormones will ultimately lead to the suppression of natural reproductive hormone effects. Hence, we refer to all processes that affect reproductive hormones as inducing hormonal incompetence [39]. There are four main modalities in this category. Firstly, disruption can be achieved through the surgical removal of gonads (gonadectomy). Secondly, disruption can be achieved through the supply of exogenous (synthetic or externally derived) versions of naturally occurring reproductive hormones (*i.e.*, testosterone, oestrogen, progestins; Table 1) that interfere with normal feedback control and secretion of endogenous hormones. This method is commonly utilised in human contraception [17,40,41]. Thirdly, endocrine disruption can be achieved by agonism or antagonism of naturally occurring (endogenous) reproductive hormones (e.g., GnRH), via the administration of chemicals that actively compete with hormone binding sites. Finally, immunocontraception can be used to induce an autoimmune response through vaccination against naturally occurring reproductive hormones (e.g., GnRH).

Physical non-endocrine methods rely on physical (rather than chemical) means to disrupt the normal functioning of the reproductive system, and do not alter naturally occurring reproductive hormone levels. These methods encompass surgical techniques that *occlude* reproductive passages (but do not remove endocrine reproductive organs) or the use of implants that physically irritate the female reproductive tract (e.g., non-hormonal intrauterine devices; [35]). Surgical techniques that occlude reproductive passages but leave endocrine reproductive function intact include modalities that ligate (oviduct ligation, vasectomy) or remove (oviduct removal, epididymectomy) reproductive passages, but not gonads (Table 1). It is important to note that traditional surgical techniques used for fertility control in companion animals remove gonads (“gonadectomy”), including castration and ovariectomy/ovariohysterectomy (Table 1), and render reproductive hormone patterns nonfunctional [42,43]. We therefore classify these methods as inducing endocrine suppression despite their application through physical means (surgery).

Chemical non-endocrine methods rely on inducing an autoimmune response through vaccination against naturally occurring ovum proteins, most commonly the zona pellucida (ZP) [44], to prevent fertilisation of ova. This is achieved in the same way that a vaccine is used to mount an immune response against a pathogenic microbe (e.g., virus or bacterium) except that the immune response is being directed towards the animal’s own ovum proteins. Once the animal’s immune system disrupts the capacity for an ovum to be fertilised, affected females are rendered infertile irrespective of how many times they are mated by a male [44]. Similarities in the effects of physical and chemical non-endocrine modalities have been demonstrated by the use of physical occlusion surgeries to model the effect of ovum protein immunocontraception in epidemiological studies [45].

This first principles approach of classifying fertility control methods according to modalities should enable more meaningful comparisons and improved assessments of welfare risks at an individual animal level. This approach should also help to clarify some misconceptions arising from the oversimplification that has been used in the past to describe classes of methods (e.g., [46]). We therefore advocate a focus on modalities, rather than colloquialisms or trade names [47], when considering the implications for individual animals for any chosen treatment.

### 2.3. Best Practice Approaches

For some time, many contentious lethal (e.g., shooting, toxins) and non-lethal (e.g., marking) wildlife management methods have been closely scrutinised, with strong focus placed upon animal welfare impacts, rather than efficacy in isolation [19,48]. Quantitative assessment methods have allowed ranking of humaneness for many lethal wildlife control methods [12], in combination with the development of “best practice” approaches (e.g., [49]). In contrast, animal welfare assessment of fertility control methods has not been evaluated with the same level of scientific rigour and much uncertainty remains regarding animal welfare impacts [50]. As identified in reviews [9], most research into welfare risks has used qualitative, anecdotal evidence, and/or presented opportunistic, qualitative data lacking appropriate controls, sampling methodology or time scales. For these reasons, reviews have identified the assessment of welfare risks for fertility control as a research priority [9]. In the absence of quantitative humaneness assessment templates for fertility control modalities, “best practice” standards have not been developed and methods are still largely chosen on an *ad hoc* basis, and there remains much variability in how modalities are chosen and applied.

## 3. Animal Welfare Risks

It is proposed that any animal treatment can have short-term adverse effects on welfare (for example those caused by an injection) followed by long-term effects, which may be very different [51]. Both should be assessed. At the same time, modern approaches to animal welfare propose that avoiding negative welfare states (harms) should be considered in addition to the provision of opportunities for pleasurable experiences (such as social play, courtship or parenting [52,53]). This approach dictates that animals need positive experiences, in addition to minimised suffering [52,54]. Below we present a list of all of the animal welfare risks associated with fertility control, from administration (short term) to the response to treatment (long term) and include those imposing negative, and restricting positive welfare outcomes, respectively.

### 3.1. Short-Term (Administration) Negative Animal Welfare State Risks

#### 3.1.1. Animal Capture, Restraint, Anaesthesia, Marking and Surgery

Most stages of wildlife management techniques that involve animal manipulation are likely to impose welfare costs. This is recognised for lethal wildlife control techniques, where animal welfare assessment frameworks require consideration of stages of animal manipulation prior to the killing method [12]. Some wildlife fertility control programs require animals to be captured and handled [55] or undergo anaesthesia and surgery [21] while other techniques allow animals to be darted (remotely treated) *in situ* [56]. Classes of fertility control methods that currently require animal capture are shown in Table 2. These restraint procedures, required to administer a fertility treatment, may impose animal welfare costs that are in addition to the physiological and behavioural effects of the fertility control procedure or agent.

**Table 2:** Animal welfare risks from administration techniques used to implement common currently used fertility control modalities in mammalian wildlife. All potential stages of animal manipulation are shown to demonstrate techniques that require multiple stages of animal manipulation and those that do not.

Mechanism	Technique	Effect of Administration					
		Capture	Anaesthesia	Surgery	Remote Administration	Multiple Doses *	Frequency of Treatment
Chemical non-endocrine	ZP immunocontraception	−	−	−	+	±	3 years
Physical non-endocrine	Vasectomy	+	+	+	−	−	Permanent
	Tubal ligation	+	+	+	−	−	Permanent
Endocrine suppression	GnRH immunocontraception	−	−	−	+	−	3 years
	GnRH agonism	±	−	−	±	−	1–2 years
	Synthetic progestins	±	−	−	±		3–5 years
	Castration	+	+	+	−	−	Permanent
	Ovariohysterectomy	+	+	+	−	−	Permanent

* 2 doses are often required to ensure the efficacy of a single treatment; ± Can be administered via remote delivery or via capture, restraint and implantation.

In contrast, other fertility control programs have recognised the potential animal welfare costs of administration procedures and have chosen methods that allow for remote delivery of a single dose of an agent [57], effectively minimising stress other than that associated with potential darting injuries [58] or injection site reactions [59]. With the use of many modalities, for example, following remote drug delivery (darting) and surgical procedures, there is a requirement for visual marking of treated animals [60,61]. Marking in itself has been demonstrated to impose animal welfare impacts in many wildlife species [48].

#### 3.1.2. Repeated Treatments

There are variations in the durations of action for different fertility control approaches. Some methods (e.g., surgical intervention) produce permanent infertility while some exogenous endocrine methods may affect fertility for less than one year (e.g., exogenous prostaglandins used to terminate pregnancy; [28]). Some chemical fertility control methods will require repeated administration every 1−2 years (e.g., GnRH agonists; [24]) or require booster administration 1−4 weeks after initial treatment (e.g., older ZP vaccines; [60]). All repeat treatments will impose additional animal welfare costs, as animals will need to be captured/injected several times. Some programs addressed the requirement for booster doses for older ZP vaccines by maintaining wild animals in captivity between injections, a process that can be highly stressful for free-ranging species [62]. For long-lived species (>5 years), multiple rounds of administration may be required to maintain infertility in treated animals [63]. An additional consideration is the aversion behaviour of wild animals after previous exposure to a stressful method. For example, studies requiring animals to be re-darted [60] or re-trapped [64] have observed greater difficulty in re-administration of a treatment.

#### 3.1.3. Injection or Implantation Site Local Inflammatory Reactions

Many remotely delivered immunocontraceptive modalities, being adjuvanted vaccines that rely on stimulating the immune system, have been shown to induce granulomatous inflammation at the site of injection [59,65]. Some studies have suggested that the welfare impact of such reactions is likely to be similar to the use of any adjuvanted vaccine. Likewise, many forms of chemical implants have demonstrated similar local inflammation effects associated with their administration [66]. The incidence of such adverse reactions, and their impact on welfare in free ranging species may be unknown, given the limited monitoring of many animals post-administration.

### 3.2. Long-Term (Treatment) Negative Welfare States Risks

#### 3.2.1. Physiological Changes

Significant or prolonged physiological changes may be considered an animal welfare concern as they may alter an animal’s ability to perform natural behaviour [67] and/or prevent adaptation to environmental challenges. Physiological responses may affect the animal directly, in terms of increased metabolic rate or redistribution of body reserves, or indirectly, in terms of loss of fitness, such as loss of appetite or decreased immunity [68]. All animals have the capacity to make behavioural changes in response to stimuli but marked physiological changes may restrict the capacity of animals to express the full range of natural behaviour [69]. Physiological changes have been demonstrated for several fertility control modalities, particularly those that derive their effect from endocrine suppression. Many studies on endocrine suppression methods have demonstrated weight gain in treated animals [25,27,70], but have considered the change to be positive or neutral, from a health perspective, without considering the effect of natural behaviour for a free-ranging species required to thermoregulate, forage, escape predators, compete with cohort animals or migrate. Immature male animals subjected to endocrine suppression generally do not grow as large as untreated males, as seen in white-tailed deer (*O. virginianus*; [71]) and tammar wallabies (*Macropus eugenii*; [66]). Endocrine suppression in immature male tammar wallabies (*M. eugenii*) also resulted in increased fat deposition [66]. Changes have also been demonstrated from endocrine suppression modalities to processes such as antler shedding in deer species [72]. Especially when endocrine suppression modalities are applied to juvenile animals, sex-specific phenotypes often fail to develop and male animals can become “feminized”. This alteration in natural development means that these impacts are permanent [66,72]. Such altered physiology will likely reduce the capacity of such animals to express a full range of normal behaviour, and adapt to environmental changes.

#### 3.2.2. Altered Behaviour

The importance of natural behaviour, and the ability of animals to experience positive welfare states, is a central tenet of modern animal welfare science [54]. Behavioural repertoires indicating satisfactory welfare should include the expression of normal patterns of maintenance and social behaviour, such as grooming, rest and play [52]. Deprivation of normal behaviour may compromise welfare by limiting an animal’s opportunities for positive experiences [52]. Changes in behaviour can be difficult to measure, but the importance of maintaining natural behaviour has been recognised for wildlife fertility control [73]. Of the few studies that have explicitly assessed behavioural changes in free-ranging wildlife (e.g., [74]), changes have been demonstrated to feeding, resting and dispersal behaviour.

For example, behavioural effects in free-ranging feral horses (*E. caballus*) subjected to endocrine suppression (GnRH immunocontraception) included observations that treated animals fed less, rested more, and travelled less [75]. Endocrine suppression in immature captive male tammar wallabies (*M. eugenii*) resulted in decreased agonistic (related to fighting) behaviour with intact males, lower dominance, and higher sexual interest from intact males [66]. Other behavioural changes are less predictable. For example, it has been demonstrated that male eastern grey kangaroos (*Macropus giganteus*) prefer to be associated with females that have not been subjected to endocrine suppression [76]. On the basis of such non-intuitive findings in an inherently complex field, we hypothesise that the real effect of altered social structures and disrupted social cohesiveness is not yet fully understood.

#### 3.2.3. Extended Breeding Seasons

Beyond any welfare impact suffered by individual animals subjected to fertility control treatment, there are also often non-intuitive welfare costs to other animals of the same species, through social interactions. Specifically, fertility control modalities that prevent pregnancy occurring without preventing oestrus behaviour (physical non-endocrine and chemical non-endocrine methods) often induce a prolonged breeding season. This phenomenon has been observed for white-tailed deer (*O. virginianus*; [4,77,78]) and feral horses (*E. caballus*; [79]). The prolongation of natural breeding seasons can represent animal welfare risks for treated and untreated animals, specifically males. For example, the study of Ji *et al.* [80] demonstrates a pronounced impact on the body condition and welfare of male brushtail possums (*Trichosurus vulpecula*) with the extension of a breeding season through the presence of sterile, but hormonally competent, females. The average body condition of these males was significantly poorer than that of males in control areas. Similar effects have been observed in male deer [81]. It has been argued that extension of reproductive cycling may have important social consequences for feral horses (*E. caballus*; [79]) and African bush elephants (*Loxodonta africana*; [82]). In addition, increases in the cumulative number of mating attempts (social contacts) with non-endocrine modalities may increase infectious disease transmission risks [45].

### 3.3. Long-Term (Treatment) Positive Welfare States Risks

#### 3.3.1. Deprivation of Parental Behaviour in Nurturing Species

Despite the central intended effects of fertility control agents in preventing the birth of offspring, and hence the opportunity for animals to express parental behaviour, the loss of this positive welfare state has not commonly been cited as a welfare concern for treated wildlife. In comparison, the welfare risks of deprivation of this natural behaviour have been recognised in zoo animals. The European Association of Zoos and Aquaria [83] stated: “limiting the opportunity to breed in species which display nurturing parental behaviour, by definition, reduces an individual animal’s opportunity to express one of the most important and complex sets of natural behaviour and can thus lead to a decrease in welfare”. Contention surrounding the importance of this deprivation has been at the heart of many discussions of ethical practices in zoos worldwide [4,84,85].

The impact of this animal welfare risk is not easily demonstrated with empirical evidence. However, deprivation of freedom to express natural behaviour is universally considered an animal welfare concern [54] as it alters an animal’s nature or “telos” [67]. Preventing such freedoms may affect animal wellbeing by reducing their ability to seek and gain pleasurable experiences and/or denying individuals the satisfaction of desires or preferences [86]. However, it must be noted that there are also welfare costs associated with reproduction, involving energetic costs and mortality risks that animals must trade-off with reproductive success [87]. Maternal behaviour in particular is complex, involving birth, nursing, and care of young. This loss of an important natural behavioural repertoire to many nurturing mammalian species warrants much greater discussion and consideration in the wildlife management sector.

#### 3.3.2. Deprivation of Courtship and Mating Behaviour

Another positive welfare state that may be deprived of animals is the freedom to express natural reproductive behaviour, including courtship and mating. Endocrine suppression modalities often reduce or eliminate courtship and mating behaviour [81], through their mechanism of action targeting endocrine processes centrally, and depressing all reproductive hormonal activity [88]. For example, white tailed deer (*O. virginianus*) males subjected to an endocrine suppression modality (GnRH immunocontraception) display very few mating attempts with untreated (hormonally competent) females [78]. Courtship and mating behaviour are considered essential components of natural behaviour for many free-ranging mammal species and the deprivation of this natural behaviour must also be considered an important animal welfare impact, whether or not it is experienced as deprivation given the lack of reproductive hormonal stimulation for the behaviour to occur.

## 4. Fertility Control in Other Species

Many fertility control modalities currently applied in wildlife management were initially developed to meet the economic and emotional needs of owners of domesticated animals, such as companion or production animals. Some endocrine suppression modalities (e.g., GnRH immunocontraception) were developed for livestock species to intentionally alter physiology (to influence meat quality) and behaviour (to reduce aggression) to benefit production, rather than to prevent reproduction [89]. Fertility control of wildlife was initially performed as an extension of the practices used by veterinarians for domestic animal control and ownership, primarily gonadectomy [42,61]. However, gonadectomy is not a universally supported practice for companion animals, and the considerable welfare costs associated with the profound endocrine suppression it induces has seen its use considered unethical or even illegal in some countries (e.g., Sweden, Norway) [42,90]. The justification of the need for routine gonadectomy of companion animals is not uniform between countries, and these inconsistent views are based on value differences [42].

In livestock management, hormonally incompetent animals have been viewed as “modified” by many scientists, with concerns about the welfare of treated animals as well as human health from consuming treated animals [51]. Many studies of domestic animals subjected to endocrine suppression modalities have demonstrated (often desired) physiological and behavioural changes, when observation methods have been intensive and long-term [89]. Despite the paucity of similar studies examining long-term changes in wildlife species, it would be naïve to assume that similar welfare costs would be unexpected in free-ranging animals that were similarly treated. It is worth considering if modalities that were originally considered humane in domestic animals should also be considered to be humane in wildlife.

## 5. Improving Assessment of Animal Welfare Risks

Animal welfare is complex and guidelines for how it should be assessed have evolved over time [91,92]. Modern approaches require that both negative and positive welfare states are considered [53], and that measures used to assess welfare states include both physiology (“health”) and behavioural measures, as well as measures of mental state [93]. Fertility control studies have primarily focused on efficacy (reproductive output) in the short term but many studies have explicitly or opportunistically assessed health side-effects [9]. Few studies have assessed broader animal welfare impacts beyond short-term health effects, such as the impact on animal wellbeing when reproductive and social behaviour patterns are disrupted in response to altered physiology, or deprivation of positive welfare states.

### 5.1. Welfare Is More than Just Health

Although animal welfare is a complex entity, the Five Domains model was proposed over 20 years ago as a conceptual framework to simplify welfare considerations for research animals [94], but it has since been extended to incorporate positive welfare states [92]. The model proposes four physical/functional domains (nutrition, environment, health, and behaviour) and the fifth domain is mental state [94].

The majority of studies that have addressed animal welfare during fertility control programs have effectively restricted their assessments to domain 3 of the model; health, as typically only measures of injury rate, the presence of disease, and body condition have been examined [59]. The high frequency with which local reactions to chemical modalities at injection/insertion sites are described in the literature [9] suggest focus on this in the past has been disproportionally high given the low intensity and duration of suffering involved. However, it has been recognised for more than a decade that assessment of animal welfare must be based on a wide range of measures in addition to health indices [95]. Community debate about intensive livestock farming has demonstrated clearly the difference between concern for animal health and welfare. While intensively raised pigs and chickens demonstrate very high health outcomes (body condition, absence of parasitism *etc.*), their welfare is considered by a large majority of the general public to be severely impaired through impedance of natural behavior, and an absence of positive experiences, that is imposed by small, restrictive housing [69,96,97].

For the majority of wildlife fertility control studies, the length of time that any side-effects have been monitored for has been restricted to short time frames (*i.e.*, 2–3 years [55,77]). However, there have also been longer term studies performed [78]. The potential for, and occurrence of, long-term behavioural changes not detected by short-term studies, has been demonstrated for marine mammal marking techniques [48], and terrestrial chemical capture techniques (bear species; [98]). These studies have urged researchers to look beyond short-term health approaches for holistic assessment of welfare. While the “short-term health” approach is deemed appropriate for assessing lethal methods where death is expected to be an acutely reached endpoint [19], it is less appropriate for non-lethal methods where duration of suffering may be chronic (years). Long-term welfare risks clearly exist for all fertility control modalities, but until evidence exists to the contrary it should be assumed that the magnitude of the impact of these effects on welfare could be significant.

### 5.2. Maintenance of Natural Behaviour in Free-Ranging Animals

Similar approaches have been used for fertility control in domestic and free-ranging animals, but there are important differences in society’s perception of our duty of care for animals in each context. There is broad acceptance that the maximisation of health should be the focus of animal welfare for the keeping of companion animals [90], while the focus of stewardship for wildlife centres on the maintenance of natural behaviour. For this reason, practices such as supplementary feeding and veterinary treatment of wildlife populations have been consistently discouraged [99,100,101], For a discussion of the role of ethics in wildlife management decisions related to health and welfare, see the study of Rolston [102].

Many fertility control methods that do affect behaviour and physiology (e.g., altered fat deposition; [103]) for companion animals are considered acceptable (by some scientists [42]) because human owners take full responsibility for the care of these animals. Decreasing a companion animal’s survival skills, such as the ability to hunt, survive adverse climatic conditions and defend itself against another competitor are deemed acceptable by society because humans can continue to provide food, shelter and protection for these animals. “Nuisance behaviour” in companion animals [104] such as fighting, roaming, territory marking, and oestrus calling, that are prevented or reduced by endocrine suppression, are all examples of natural behaviour essential for the welfare of free-ranging animals. This distinction between the behavioural needs of companion and free-ranging animals has not been adequately recognised by authors studying species that may exist in either state (e.g., feral/domestic cats (*F. catus*); [103,104]). We accept that the management of some free-ranging species blurs the line between domestic and wild animals (e.g., zoo animals; [84], or animals partially confined in fenced urban reserves; [21]). For these contexts, preservation of natural behaviour may be argued to be ethically less important if animals are not required to escape predation, or extensively forage. This consideration is particularly important given that many studies attempting to assess welfare impacts of fertility control modalities have provided *ad libitum* access to resources (water and feed) for study animals [105,106]. In some contexts where animals are not considered clearly domestic *or* wild (e.g., “street dogs” [42,61]), some management programs have explicitly aimed to modify natural behaviour when it creates conflict with human values (e.g., aggression leading to attacks on humans [61]).

The same logic should not apply for all wild animals, as the duty of care provided by humans often ends for managed wildlife at the point of release from restraint or administration of treatment (e.g., [107]). For this reason in feral horse (*E. caballus*) fertility control, approaches that impact upon androgenic (male) behaviour [26] are no longer considered humane or appropriate. In another context, it has been argued that the only fertility control options that should be considered for coyotes (*Canis latrans*) should be those that allow the animals to remain hormonally competent, such is the importance of the endocrine system for the maintenance of natural behaviour [108]. We argue that the preservation of hormonal competence is important to maximise the welfare of wild animals, due to the profound effects of endocrine suppression on important behaviour such as mating, aggression and dominance.

## 6. Case Studies

To illustrate the dissimilarities in approaches that have been taken to fertility control in different species and jurisdictions, we present two case studies. The first describes refinement of modalities used for feral horses (*E. caballus*) in North America and the second describes the relative lack of refinement in modalities considered suitable for Eastern grey kangaroos (*M. giganteus*) in Australia.

### 6.1. Case Study: Feral Horses (E. caballus) in North America

Fertility control modalities for feral horses have been investigated for several decades in the USA [26,109]. While a range of modalities were used in the past (e.g., exogenous testosterone; [26], intrauterine devices; [35]; Table 1), more recently, remotely delivered GnRH immunocontraception [110] and particularly ZP immunocontraception [62,74,79,111], have become the focus. Importantly, it was recognised decades ago that the short-term welfare risks associated with immobilisation and capture necessitated that fertility control modalities were administered remotely [56]. Immunocontraception vaccines may be administered via remote delivery, removing the need for capture, anaesthesia or surgery, but the requirement for booster administration with older ZP vaccines led some authors to maintain horses in captivity between treatments [62]. However, ZP vaccines also often lead to extended breeding seasons, and therefore there are accompanying increased energetic costs and injury risks from increased mating attempts [112]. The focus on ZP immunocontraception developed from a formal process initiated by the Bureau of Land Management (BLM) [111] in the USA to identify the most humane and practical fertility manipulation options. This departure from *ad hoc* trials of many different methods has seen progress towards adoption of “best practice” methods.

### 6.2. Case Study: Eastern Grey Kangaroos (M. giganteus) in Australia

Many different fertility control modalities have been trialed for easily captured eastern grey kangaroos in Australia [20]. The fertility control modalities that have been applied to kangaroos and their accompanying welfare risks have recently been reviewed [113]. Much recent research has focused on the use of a range of endocrine suppression modalities. GnRH agonists [24,105], and synthetic progestins [20,63,114], have been the subject of several studies. Chemical non-endocrine (ZP immunocontraception; [115]) and physical non-endocrine (vasectomy; [21]) modalities have also been investigated less frequently. However, eastern grey kangaroos are still managed with physical endocrine suppression modalities (castration; [21]) that are rarely applied to other wildlife species. In addition, modalities that have been delivered via remote injection in other contexts (e.g., GnRH agonists; [116]) are often delivered to eastern grey kangaroos as implants, following capture, restraint and anaesthesia ([105]). There have been no formal attempts to standardise modalities (but see [113]), and no apparent progress toward development of “best practice” guidelines for kangaroo fertility control in Australia, and so *ad hoc* methods continue to be used without careful consideration of animal welfare impacts.

The management of Australian eastern grey kangaroos contrasts strongly with the management of American feral horses. In the USA, management goals have included minimising animal welfare impacts of fertility control on wildlife, particularly feral horses [4], by using methods that only rely on darting animals. In Australia, marsupials are still regularly caught, and subjected to anaesthesia and surgery to administer fertility control treatments that, when compared to ZP immunocontraception, have greater effects on their natural behaviour and physiology [21,63,66]. In Australia, similar modalities have not only been used in several kangaroo species [24], but also wallaby species [66,117] and koalas (*Phascolarctos cinereus*) [27,115,118].

These case studies have shown the different approaches taken towards fertility control in two wildlife contexts and highlight the need for increased consideration for the welfare risks of current practices. For this reason, we propose a framework to better enable researchers and managers to understand the animal welfare risks of any fertility control modality and to improve the balance between meeting animal interests with management needs.

## 7. Suggested Future Improvements

Given the several decades over which wildlife fertility control research has been performed, we contend that sufficient information exists to move from a *carte blanche* approach towards a “best practice” approach for refining methods to minimise animal welfare impacts. It is our contention that methods that induce endocrine suppression, and requiring repeated or multi-stage interventions, are likely to represent poorer animal welfare outcomes than those methods that do not (Table 3). We suggest where duration is similar, the modality inducing the least changes to natural behaviour and physiology will pose the least welfare risk. Likewise for administration risks, the total welfare risk should be considered as the cumulative total of the invasiveness of the procedure, and the duration and frequency of the procedure. For this reason, we propose that welfare risks associated with long-term treatment effects are considered a higher welfare priority than short-term administration risks.

We contend that the deprivation of parental behaviour is an unavoidable welfare cost common to all fertility control modalities. Beyond this cost, several studies have identified that the modalities that minimise welfare risks prevent pregnancy but do not disrupt endocrine function, thus ensuring that reproductive/social behaviour changes are minimised [39,79]. However, with the availability and marketing of modalities that induce endocrine suppression but have appealing ease of administration benefits (e.g., GonaCon™), many researchers have opted to induce endocrine suppression in treated animals despite the existence of alternative modalities that do not (e.g., ZP immunocontraception). We recognise that GnRH vaccines offer increased efficacy and convenience through their effectiveness against both sexes, single injection administration, and lack of extended breeding seasons. However, these efficacy advantages should be balanced against clearly increased welfare costs for a method that causes changes to natural behaviour and physiology [89].

**Table 3:** Suggested principles for assessing cumulative animal welfare impacts for wildlife fertility control modalities. Methods are listed from top (least animal welfare impact) to bottom (most impact). For example, administration methods requiring capture, anaesthesia, and surgery are given a +++ rating. ZP = zona pellucida. GnRH = gonadotrophin releasing hormone.

Mechanism	Technique	Impact of Treatment	Impact of Administration
Chemical non-endocrine	ZP immunocontraception	+	+
Physical non-endocrine	Vasectomy	+	+++
	Tubal ligation	+	+++
Endocrine suppression	Synthetic progestins	++	++
	GnRH agonism	++	++
	GnRH immunocontraception	+++	+
	Gonadectomy	+++	+++

We suggest that methods requiring capture, anaesthesia, and/or surgery, or frequently repeated administration (in long-lived species) represent poorer animal welfare outcomes when compared to remotely deliverable methods (Table 3). Given the above animal welfare risks associated with treatment (long-term) and administration (short-term), we contend that remotely deliverable non-endocrine vaccines (*i.e.*, ZP immunocontraception) are likely to be the most humane fertility manipulation methods currently available (Table 3). While welfare impacts are high for administration of physical non-endocrine modalities (vasectomy, tubal ligation), repeated treatments are not required and hormonal competence is maintained. For all non-endocrine modalities, important positive welfare states (courtship, mating) are maintained. However, the effects of extended breeding season associated with all non-endocrine modalities suggests that species specific traits need to be considered to avoid welfare impacts relating to large increases in mating attempts (e.g., [80]) or the birth of offspring in non-favourable seasons [78].

On the same mechanistic basis, we contend that gonadectomy represents the least humane of the currently available fertility control technique for wildlife. Gonadectomy requires capture, often major (abdominal) surgery, anaesthesia, and induces complete and irreversible reproductive endocrine suppression, and accompanying permanent changes in natural behaviour and physiology [42,90]. We suggest that the welfare impacts associated with gonadectomy render it appropriate only for domestic animals where a human duty of care is provided, and where behavioural changes are desired (e.g., [119]). GnRH immunocontraception and agonism both induce partial suppression of hypothalamic-pituitary-gonadal axis function [106], but the effects are not as profound as those of gonadectomy. GnRH immunocontraception is likely to pose the lowest welfare risks of all endocrine suppression modalities due to its’ favourable administration method (Table 3). GnRH agonism and exogenous progestin modalities both induce endocrine suppression and require repeated administration (sometimes via remote delivery but often requiring capture), but not major surgery (Table 3). However, there has been progress toward developing remote delivery systems for both progestin implants [56] and GnRH agonists [116], reducing the short-term welfare risks associated with their administration.

### Suggested Principles for Selection of Fertility Control Methods

In an attempt to improve the present approach to the choice of fertility approaches, some authors have suggested the use of formal criteria for the selection of methods that are appropriate for field applications [2]. While we support the criteria approach of Massei and Cowan [2] for assessing efficacy, we suggest that more holistic scientific rigour should be applied to assessing welfare impacts. We present Table 3 as a framework for assessing the welfare pros and cons of commonly used modalities. The challenging nature of making welfare decisions does not justify averting from the central tenets of scientific logic: observation, hypothesis and experiment. Many decisions on the choice of fertility control modalities have been *carte blanche* and this represents the antithesis of scientific reasoning. We have attempted to address the short-term and long-term animal welfare risks, as per Broom [51], in a framework using a weighting approach (as per Dubois and Fraser [101]). Table 3 summarises the parameters that we consider most important for selecting control methods, and we suggest the following principles for selecting the most humane fertility control methods for wildlife populations:

(1) It should be recognised that all fertility control methods impose animal welfare impacts. The deprivation of the opportunity to display parental behaviour in nurturing species is an animal welfare impact,
(2) Methods should be chosen that pose the lowest cumulative animal welfare cost, including capture, administration and frequency of treatment,
(3) All domains of animal welfare should be considered, including behaviour and the opportunity for animals to have positive experiences,
(4) Methods that maintain hormonal competence, thus promoting natural behaviour, should be chosen over methods that induce endocrine suppression, and
(5) Where uncertainty exists regarding welfare impacts, fertility control modalities should not necessarily be chosen over alternative lethal or non-lethal methods.

Any decisions regarding wildlife management will involve seeking a balance between the interests of the wildlife and other stakeholders. The suggested use of our framework in ethical decision-making has some unavoidable subjectivity relating to the difficulty in comparing various types of risks (e.g., long duration-low intensity welfare impacts with short duration-high intensity welfare impacts), which value-based ranking systems (e.g., [12]) may address. However, we contend that the proposed weighting approach will be more informative for wildlife managers than a list of comparative features without commentary. In addition, the weighting approach has been widely applied for welfare studies lacking comprehensive empirical evidence of all aspects (e.g., [101]).We present this framework as a provocation to wildlife researchers and wildlife managers to improve the rigour with which they assess management options, and to broaden their understanding of animal welfare.

We recognise that the range of species and contexts for which wildlife fertility control is desired makes the proposal of a single preferred method impossible [40]. We accept that efficacy, economic costs, ecological side-effects, and other factors are also important considerations for fertility control programs [2]. We present this framework to help with objective assessment of animal welfare, and to aid managers in achieving balance when weighing welfare impacts against other management considerations. It is an accepted reality that methods representing optimal animal welfare outcomes may not be appropriate for all management contexts. We contend that understanding the complexities of both reproductive physiology and animal welfare science should be improved in wildlife management. Claims that fertility control modalities pose “no undesirable physiological or behavioural side-effects” from limited observations [105] are unrealistic and display a poor understanding of animal welfare. Likewise, the lack of consideration for the importance of depriving animals of positive experiences when comparing fertility control to other management options requires re-consideration. The lack of standardisation of wildlife fertility control modalities in many contexts is indicative of the enduring misconception that non-lethal reproductive manipulation is synonymous with benign outcomes. We contend that wildlife fertility control should not always be assumed to be humane.

## 8. Conclusions

Efforts to apply fertility control to wildlife have now been performed for over 30 years but there has been little standardisation of procedures over this time. Research into this area has primarily focused on the important aspect of efficacy but this has meant that the associated impacts on animal welfare have only seldom been addressed. Consequently, there is a growing need for more meaningful interchange between animal welfare scientists, wildlife biologists and wildlife managers [120]. Understanding the mechanisms of fertility control modalities and the resultant impacts on natural behaviour are important and have been under-appreciated. Broader understanding of modern animal welfare science and the importance of positive welfare indicators are required for wildlife managers. We contend that endocrine suppression in free-ranging animals is an undesirable outcome because of its long-lasting alterations to natural behaviour and physiology. Not all fertility control modalities induce endocrine suppression and these methods that do not should be treated preferentially. Likewise, administration methods that require capture, surgery, or frequently repeated treatment are similarly undesirable. Animal welfare impacts should be recognised and carefully balanced against existing management goals to ensure continuous improvement in wildlife fertility control methods.
